# Cytokine Inhibitors Upregulate Extracellular Matrix Anabolism of Human Intervertebral Discs under Alginate Beads and Alginate-Embedded Explant Cultures

**DOI:** 10.3390/ijms241512336

**Published:** 2023-08-02

**Authors:** Kenichiro Kakutani, Takashi Yurube, Howard S. An, Minoru Doita, Koichi Masuda

**Affiliations:** 1 Department of Orthopaedic Surgery, Kobe University Graduate School of Medicine, 7-5-1 Kusunoki-cho, Chuo-ku, Kobe 650-0017, Japan; takayuru@med.kobe-u.ac.jp; 2 Department of Orthopaedic Surgery, Rush University Medical Center, Orthopaedic Building, Suite 300, 1611 W Harrison Street, Chicago, IL 60612, USA; howard.an@rushortho.com; 3 Department of Orthopedic Surgery, Iwate Medical University School of Medicine, 2-1-1, Idaidori, Yahaba-cho, Showa-gun, Iwate 028-3895, Japan; doita@iwate-med.ac.jp; 4 Department of Orthopaedic Surgery, University of California, San Diego, 9500 Gilman Dr. Mail Code 0863, La Jolla, CA 92093-0863, USA; komasuda@health.ucsd.edu

**Keywords:** cytokine, intervertebral disc metabolism, autocrine

## Abstract

We investigated the effects of the cytokine inhibitors IL-1 receptor antagonist (IL-1Ra) and soluble tumor necrosis factor receptor-1 (sTNFR1) on the extracellular matrix metabolism of human intervertebral discs (IVDs) and the roles of IL-1β and TNF in the homeostasis of IVD cells. The 1.2% alginate beads and the explants obtained from 35 human lumbar discs were treated with cytokine inhibitors. Extracellular matrix metabolism was evaluated by proteoglycan (PG) and collagen syntheses and IL-1β, TNF, and IL-6 expressions after three days of culture in the presence or absence of IL-1Ra, sTNFR1, and cycloheximide. Simultaneous treatment with IL-1Ra and sTNFR1 stimulated PG and collagen syntheses in the NP and AF cells and explants. The IL-1β concentration was significantly correlated to the relative increase in PG synthesis in AF explants after simultaneous cytokine inhibitor treatment. The relative increase in PG synthesis induced by simultaneous cytokine treatment was significantly higher in an advanced grade of MRI. Expressions of IL-1β and TNF were upregulated by each cytokine inhibitor, and simultaneous treatment suppressed IL-1β and TNF productions. In conclusion, IL-1Ra and sTNFR1 have the potential to increase PG and collagen synthesis in IVDs. IL-1β and TNF have a feedback pathway to maintain optimal expression, resulting in the control of homeostasis in IVD explants.

## 1. Introduction

Low back pain is a major cause of disability and is the leading cause of years lived with disability, which has a significant socioeconomic impact [1]. More than 75% of people experience low back pain in their lifetime. Low back pain restricts activity, and 15–20% of people require medical care for this malady [2]. Moreover, it leads to significant economic losses, including an annual average of 297 million restricted days and 87 million disability days in the United States. Consequently, resolving low back pain is an urgent matter worldwide. 

Intervertebral disc (IVD) degeneration may be a major factor in the pathogenesis of low back pain and lumber disc herniation. Structurally, the IVD consists of an outer annulus fibrosis (AF), which is rich in collagens that account for its tensile strength, and an inner nucleus pulposus (NP), which contains large proteoglycans (PGs) that retain water to resist compressive forces [3,4,5]. Biologically, disc cells residing in the AF and NP actively regulate the homeostasis of IVD explants by maintaining a balance between anabolism and catabolism. Although the exact pathogenesis is not well understood, IVD degeneration is considered to be a pathological condition induced mechanically and mediated biologically and is often concurrent with aging. Inflammatory cytokines, such as ILs and TNF, have been observed in degenerated IVDs [6,7]. Disc cell metabolism is regulated by various molecules, such as cytokines, enzymes, enzyme inhibitors, and growth factors, which act in paracrine and/or autocrine fashions [8,9,10,11,12,13]. IVD degeneration often results from an imbalance between anabolic and catabolic processes or from a loss of steady-state metabolism that is maintained in the normal disc. Polypeptide growth factors play important roles in anabolic regulation, which include insulin-like growth factor-1 (IGF-1), transforming growth factor-β, and bone morphogenetic proteins (BMPs) [9,14]. The IVD expresses cytokines, including interleukin (IL)-1 and tumor necrosis factor (TNF)-α. In degenerated or herniated IVD explants, the expression of these cytokines increases at both protein and mRNA levels [15,16]. The autocrine production of cytokines is an important regulatory mechanism of cartilage metabolism. Although IL-1 affects both anabolic and catabolic pathways in the IVD, IL-1 at low concentrations is more effective at inhibiting aggrecan synthesis than at stimulating its degradation. Le Maitre et al. [17] reported that the expression of IL-1β increases in human degenerated or herniated IVD cells at protein and mRNA levels. They emphasized that IL-1β is a therapeutic target to restrain catabolic pathways in degenerated IVDs. Seguin et al. [18] stated that TNF expressed by NP cells of degenerated IVDs may contribute to degenerative changes in disc diseases. IL-1β regulates the expressions of IL-1β and TNF in an autocrine fashion [19]. The expressions of some cytokines have been suggested to be modulated by negative and/or positive feedback systems. However, the function of the cooperative interplay between IL-β and TNF in the pathomechanism of IVD degeneration is unclear.

We hypothesized that the interplay of these cytokines produced in autocrine and/or paracrine fashions suppresses the basal synthesis level of matrix molecules. The aim of this study was to investigate the production of proinflammatory cytokines in human IVD cells and to reveal the role of these cytokines in the ECM metabolism of IVD cells using cytokine inhibitors IL-1 receptor antagonist (IL-1Ra) and soluble TNF receptor-1 (sTNFR1).

## 2. Results

### 2.1. Expression of Cytokines in the IVD 

#### 2.1.1. Basal Cytokine Production by Disc Explants

In half of the donors, IL-1β, TNF, and IL-6 were not detected in conditioned media from the alginate bead culture system at 2 days of preculture. Because several reports identified the presence of cytokines in the ECM compartment of the IVD [17], we measured the concentrations of these cytokines in conditioned media from the explant culture system. The values obtained from the explant culture system were normalized to the DNA content in each sample. IL-1β, TNF, and IL-6 concentrations were extremely low, and some samples of the eight donors were negative in high-sensitivity ELISAs. These data are presented in the Table 1. Each donor produced various basal amounts of cytokines in explant culture during the first 2 days of culture. No significant relationships were observed between MRI, age, and gender and the IL-1β, TNF, and IL-6 concentrations.

#### 2.1.2. Source of Cytokines in the ECM Compartment of IVDs (Disc Explants)

**Figure 1:**
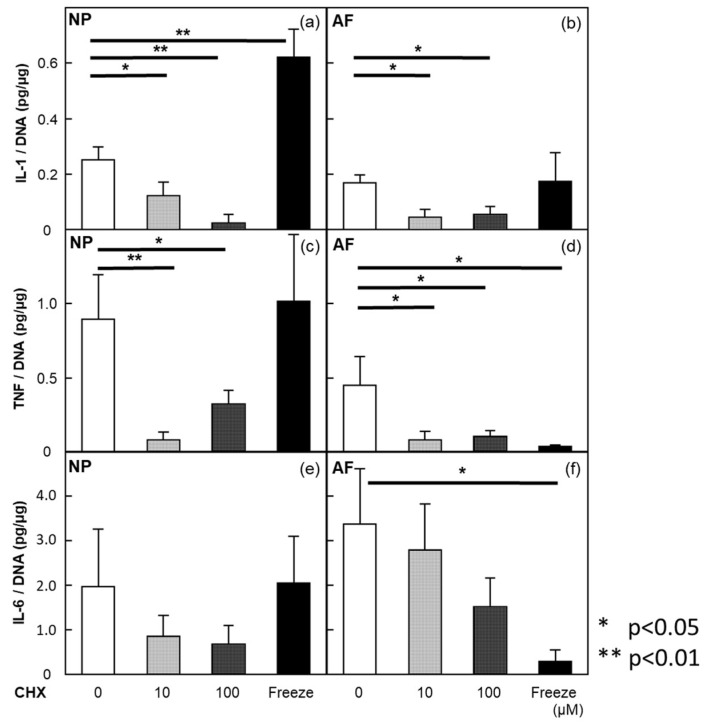
Cytokine levels in condition medium after cycloheximide treatment. (**a**,**b**) In NP and AF explants, IL-1β was significantly diminished by treatment with cycloheximide. In NP explants, freezing and thawing of cells and following incubation in medium induced the significant release of IL-1β. (**c**) In NP explants, TNF was significantly diminished by treatment with cycloheximide. (**d**) In AF explants, cycloheximide and the freezing and thawing of cells decreased TNF. (**e**) In NP explants, IL-6 showed a similar trend to TNF but did not achieve statistical significance. (**f**) In AF explants, IL-6 was significantly reduced by freezing and thawing. All values are expressed as the mean ± SE. * *p* < 0.05; ** *p* < 0.01. Data were obtained from five donors.

In NP and AF explants, IL-1β expression was significantly diminished by treatment with 10 and 100 µM cycloheximide. In NP explants, the freezing and thawing of cells and following incubation in media induced a significant release of IL-β (Figure 1a). However, in NP explants, TNF expression was significantly diminished by treatment with 10 and 100 µM cycloheximide (Figure 1c). In AF explants, cycloheximide and the freezing and thawing of cells decreased TNF expression, which was in contrast to IL-β (Figure 1b,d). In terms of IL-6 expression, in NP explants, the results showed a similar trend to TNF but did not achieve statistical significance (Figure 1e). However, in AF explants, IL-6 expression was significantly reduced by freezing and thawing, and IL-6 level, after treatment with cycloheximide, was maintained at the same level as the control. The IL-1β production suggested that the release of cytokines reflected newly synthesized cytokines or the release of intracellular cytokines induced by a newly synthesized protein, the IL-1-converting enzyme. Additionally, NP explants had a large amount of IL-1β in the intracellular compartment. In terms of TNF expression, in NP explants, the same trend as IL-1β was observed. In AF explants, TNF might be released from other explants and induced by the newly synthesized TNF-converting enzyme. IL-6 was produced in NP explants and released from other explants among AF explants. As mentioned above, IL-1β, TNF, and IL-6 were probably produced from NP and AF explants in autocrine and/or paracrine fashions. These results were obtained from five donors. 

### 2.2. DNA Concentration in the IVD

**Figure 2:**
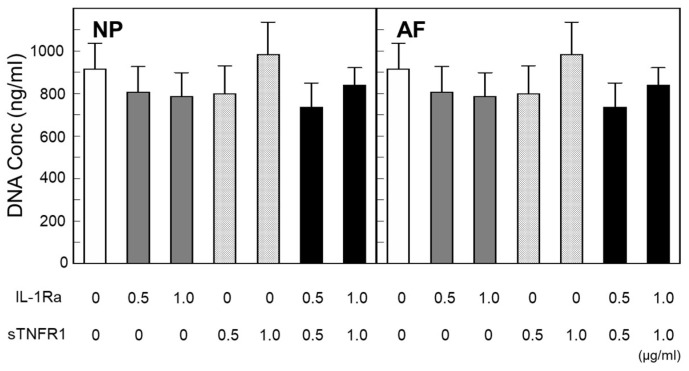
DNA concentration in NP and AF. Data were obtained from six donors.

In NP and AF explants, DNA concentrations showed that the trend decreased from the treatment of IL-1Ra (0.5, 1.0 μg/mL) and/or sTNFR (0.5, 1.0 μg/mL) but did not achieve statistical significance (Figure 2). 

### 2.3. Effect of Cytokine Inhibitors on Anabolism of the ECM Compartment in IVDs Alginate Bead Culture 

The effect of cytokine inhibitors on PG and collagen synthesis was evaluated by the ratio normalized to the value of the control group.

#### 2.3.1. PG Synthesis (Figure 3a,b)

**Figure 3:**
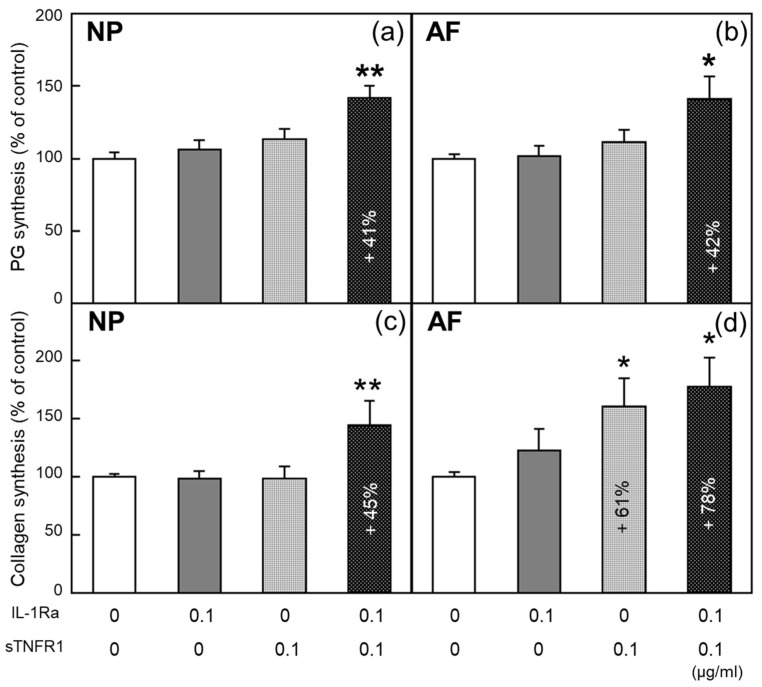
PG and collagen syntheses after treatment with cytokine inhibitors IL-1Ra and sTNFR1 in the alginate bead culture system. A single treatment with IL-1Ra or sTNFR1 did not affect PG synthesis in the both cell types. However, PG synthesis was significantly stimulated by simultaneous treatment with IL-1Ra and sTNFR1 at 0.1 ng/mL in NP cells (**a**). In AF cells, PG synthesis stimulated by simultaneous treatment showed the similar result (**b**). Collagen synthesis was significantly upregulated by simultaneous treatment at 0.1 µg/mL in NP cells (**c**). In AF cells, collagen synthesis was significantly stimulated by a single treatment with 0.1 µg/mL sTNFR1 and simultaneous treatment at 0.1 ng/mL (**d**). All values are expressed as the mean ± SE. * *p* < 0.05; ** *p* < 0.01. Data on PG synthesis and collagen synthesis were obtained from 15 and 9 donors, respectively.

In NP and AF cells, a single treatment with IL-1Ra or sTNFR1 at 0.1 µg/mL did not affect PG synthesis. In NP cells, simultaneous treatment with IL-1Ra and sTNFR1 at 0.1 µg/mL significantly upregulated PG synthesis (+41%, *p* < 0.01, Figure 3a). Additionally, the single and simultaneous treatments at any other concentration did not stimulate PG synthesis. In AF cells, the results from single treatments showed a similar trend to NP cells with a statistical significance (Figure 3b). These results were obtained from 15 donors.

#### 2.3.2. Collagen Synthesis (Figure 3c,d) 

In NP cells, a single treatment with IL-1Ra or sTNFR1 at any concentration did not affect collagen synthesis, whereas simultaneous treatment at 0.1 µg/mL significantly increased collagen synthesis (+45%, *p* < 0.01). In AF cells, a single treatment with 0.1 µg/mL sTNFR1 significantly stimulated collagen synthesis (+61%, *p* < 0.05), and simultaneous treatment at 0.1 µg/mL significantly increased collagen synthesis (+78%, *p* < 0.05). These results were obtained from seven donors.

### 2.4. Effect of Cytokine Inhibitors on Anabolism of the ECM Compartment in IVD Explant Culture

Because several reports observed the presence of some cytokines in the extracellular matrix of spinal discs, we used the explant culture system to reflect in vivo conditions. The explants were obtained from one disc. NP explants were dissected from the center of the disc, and AF cells were isolated from the anterior portion of the disc. The samples were obtained from eight cadavers, and the information is shown in the Table 1.

#### PG Synthesis (Figure 4)

The effect of cytokine inhibitors on PG synthesis was assessed by the relative ratio compared with the value of the control group, which was normalized to the DNA content in each sample. In NP and AF explants, a single treatment with IL-1Ra or sTNFR1 at any concentration did not affect PG synthesis. Simultaneous treatment with IL-1Ra and sTNFR1 at 1 µg/mL significantly stimulated PG synthesis (NP: +96%, *p* < 0.01; AF: +44%, *p* < 0.05). Moreover, the recovery rate was higher than that in the alginate bead culture. These results suggested that IL-1β and TNF in the ECM compartment participated in modulating ECM anabolism in IVD cells. These results were obtained from eight donors.

**Figure 4:**
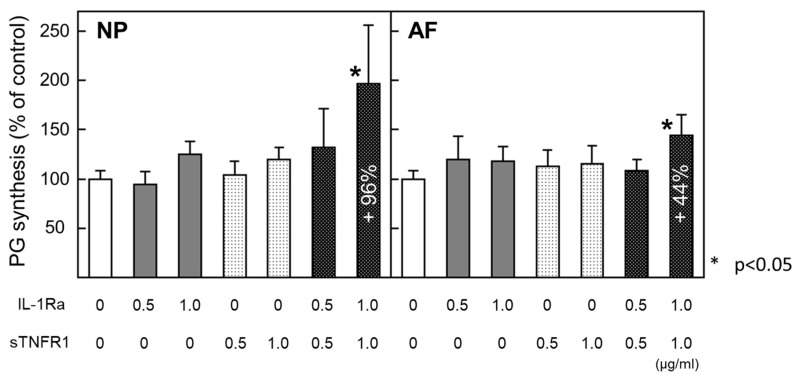
PG synthesis after treatment with cytokine inhibitors IL-1Ra and sTNFR1 in the explant culture system. Simultaneous treatment with IL-1Ra and sTNFR1 at 1.0 ng/mL significantly stimulated PG synthesis in both NP and AF cells. All values are expressed as the mean ± SE. * *p* < 0.05. Data were obtained from eight donors.

### 2.5. Relationship between Cytokine Levels or IVD Degeneration and the Effect of the Cytokine Inhibitors on PG Synthesis (Figure 5)

**Figure 5:**
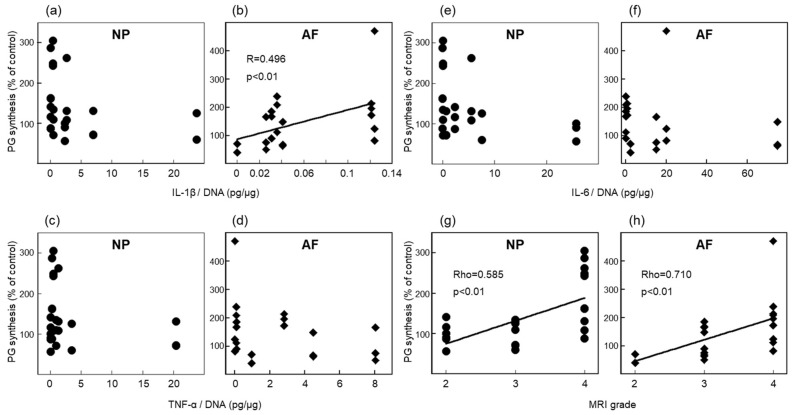
Relationship between the response to cytokine inhibitors and cytokine concentrations or the MRI grade in the explant culture system. (**a**,**b**) The concentration of IL-1β was significantly correlated to a relative increase in PG synthesis after simultaneous cytokine inhibitor treatment at 1 μg/mL in AF explants. The concentrations of TNF (**c**,**d**) and IL-6 (**e**,**f**) showed no relationship with PG synthesis. (**g**,**h**) The relative increase in PG synthesis by simultaneous cytokine treatment was significantly higher in an advanced grade of MRI. Both were significantly correlated. Data were obtained from eight donors.

Approximately one-third of donors did not show any upregulation in PG or collagen syntheses by cytokine inhibitors in alginate and explant culture systems. Next, we found a relationship between the basal levels of cytokines or the degree of IVD degeneration and the response to cytokine inhibitors by ECM anabolism. Cytokine levels were measured in conditioning media obtained after preculture from the explant culture system by ELISAs. The IL-1β concentration had a significant positive correlation to the response to simultaneous cytokine inhibitor treatment at 1 µg/mL in AF explants (R = 0.496, *p* < 0.01, Figure 5b). AF explants that produced a high level of IL-1β responded more to simultaneous treatment in terms of PG synthesis, compared with those that produced less IL-1β. However, in NP explants, no correlation was observed between the IL-1β level and the response to cytokine inhibitors. Neither the expression of TNF nor IL-6 in NP and AF explants had any correlation to PG synthesis after treatment with various concentrations of cytokine inhibitors. A relative increase in PG synthesis after simultaneous cytokine treatment was significantly higher, with an advanced MRI grade. Both NP and AF explants were significantly correlated (NP: Rho = 0.585, *p* < 0.01; AF: Rho = 0.710, *p* < 0.01, Figure 5g,h). These results were obtained from eight donors.

### 2.6. Cytokine Expression after Treatment with Cytokine Inhibitors (Figure 6) 

**Figure 6:**
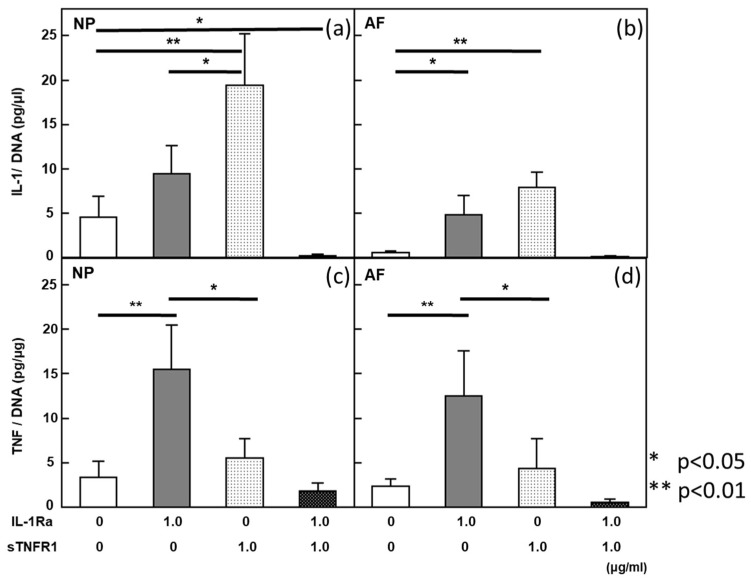
Cytokine levels in condition medium after cytokine inhibitor treatment. (**a**,**b**) IL-1β expression was stimulated by a single treatment with IL-1Ra or sTNFR1. sTNFR1 significantly increased IL-1β expression more than IL-1Ra in NP and AF. (**c**,**d**) Simultaneous treatment suppressed IL-1β production in both NP and AF. IL-1Ra significantly stimulated TNF expression. Simultaneous treatment did not affect TNF production. All values are expressed as the mean ± SE. * *p* < 0.05; ** *p* < 0.01. Data were obtained from eight donors.

IL-1β expression in AF explants had a significant positive correlation to the response to cytokine inhibitors. Because simultaneous treatment stimulated PG synthesis in the explant culture system, the interplay between IL-1β and TNF appeared to control the basal level of ECM metabolism in the IVD. The concentrations of these cytokines on day one after treatment with cytokine inhibitors were measured in a conditioning medium from the explant culture system. The cytokines were maintained at the same levels for three days after treatment. The IL-1β concentration was significantly decreased by simultaneous treatment at 1 µg/mL in NP and AF explants (Figure 6, top panel). However, IL-1β production was significantly upregulated by a single treatment with IL-1Ra or sTNFR1 at 1µg/mL, and the amount of IL-1β upregulated by sTNFR1 was larger than that upregulated by IL-1Ra in both explants. In terms of the TNF concentration, simultaneous treatment resulted in a trend similar to that of IL-1β in NP and AF explants. These results did not show a significant difference. However, a single treatment with IL-1Ra significantly stimulated TNF expression, and a single treatment with IL-1Ra significantly increased TNF expression more than sTNFR1 expression in both explants. IL-6 production was significantly diminished by simultaneous treatment with IL-1Ra and sTNFR1. These results suggested that low levels of IL-1β, TNF, and IL-6 were maintained by each feedback pathway via IL-1β and TNF signaling in degenerated IVD cells. These results were obtained from eight donors.

## 3. Discussion

Low back pain is a significant problem in society, and a major cause is IVD degeneration [3,20]. The degeneration mechanism in IVD tissues remains unclear. IL-1 might play a major role in the pathogenesis of human disc degeneration [17,21,22]. Kandel et al. [18] found that TNF might also contribute to IVD degeneration by examining TNF-induced NP explant degradation. Le Maitre et al. [23] reported the use of IL-1Ra gene therapy to inhibit IVD degeneration. Simultaneous blockage of IL-1 and TNF is effective against matrix degradation of articular chondrocytes [24,25,26]. However, no report has investigated IL-1 or TNF inhibitors for stimulating ECM anabolism of IVDs at the protein level. Our results demonstrated that IL-1Ra and sTNFR1 had the potential to increase PG and collagen syntheses in degenerated IVD tissues. Except for the stimulation of collagen synthesis by a single treatment with sTNFR1 in the AF from the alginate bead culture system, a single treatment with IL-1Ra or sTNFR1 did not affect the ECM anabolism of IVD cells. However, simultaneous treatment with IL-1Ra and sTNFR1 stimulated ECM anabolism in both cells and both culture systems. Additionally, the stimulated product by simultaneous treatment of the explant culture system was higher than that of the alginate bead culture system. Because cytokines in the ECM might be removed during the preparation process of alginate beads, these cytokines might act on the ECM anabolism of degenerated IVD cells. 

IL-1β, TNF, and IL-6 productions in degenerated IVD cells were relatively low. These cytokines were probably produced by NP and AF explants in autocrine and/or paracrine fashions. A single treatment with IL-1Ra or sTNFR1 upregulated each cytokine production, and simultaneous treatment diminished their production. No previous study has investigated the function of IL-1β in the ECM anabolism of IVD cells. Degenerative changes in IVDs are caused by an imbalance in catabolic and anabolic activities. Certain growth factors stimulate anabolism, and proinflammatory cytokines activate catabolic pathways. IL-1 knockout mice exhibit normal growth [27]. After surgery to induce OA joints, IL-1β knockout mice show accelerated development of OA lesions in both operated and unoperated joints [28]. IL-1 may not be a major factor in OA, at least in the early stages when inflammation is absent. IL-1 and TNF activate specific mitogen-activated protein kinase (MAPK) subgroups, including ERK1/2, c-Jun, and p38, in human articular chondrocytes [29]. Fukui et al. [30] reported that IL-1 and TNF stimulated BMP-2 expression in normal and osteoarthritic chondrocytes. Blocking p38 MAPK increases the mRNA expression of matrix proteins (e.g., aggrecan, collagen I, and collagen II) and anabolic factors, including insulin-like growth factor-1, transforming growth factor, and SOX-9 in rabbit NP cells [31]. Depending on degeneration, age, and cytokine concentrations in IVD tissues, the function of proinflammatory cytokines in the ECM metabolism of IVD tissue appears to alter dramatically. In addition to the crucial roles of IL-1 and TNF in catabolic pathways, these proinflammatory cytokines participate in anabolic pathways of ECM metabolism in IVDs. Thus, IL-1 and TNF may act as major regulators of homeostasis in ECM metabolism. 

Each donor produced various basal amounts of cytokines in the organ culture. We hypothesized that proinflammatory cytokines modulated the basal level of ECM synthesis and the rate of catabolic pathways in autocrine and/or paracrine fashions, which was supported by our results. However, despite some donors with high levels of these cytokines, they did not respond to treatment with the cytokine inhibitors alone or in combination. Pathways other than IL-1β and TNF might affect the anabolism of IVDs. Some donors responded strongly to the treatment on PG synthesis, even if they had low levels of these cytokines. Except for the significant relationship between IL-1β expression and the relative increase in PG synthesis in AF explants, no significant relationships between cytokines IL-1β, TNF, and IL-6 and PG synthesis were observed in NP and AF explants. The relative increase in PG synthesis induced by simultaneous cytokine inhibitor treatment was significantly higher in an advanced MRI grade in both NP and AF. The MRI grade in an IVD might reflect the degenerative change in the ECM compartment in IVDs. Furthermore, the various and optimal levels of proinflammatory cytokines might modulate basal anabolism in each donor and each stage of degeneration in IVD cells. AF was especially dense, compared to NP. We could not deny the possibility that explant culture limited the diffusion of cytokines; further investigations are required.

Studies have reported the autocrine fashions of IL-1 and TNF in human articular chondrocytes [19,32,33]. In this study, the absence of IL-1 and TNF pathway signaling upregulated the expressions of each other. Basal cytokine levels could be maintained by the interplay of IL-1 and TNF in degenerated IVD explants via a feedback system. As the largest avascular explant, ECM metabolism of IVDs was restrained by low pH and low oxygen tension. IL-1 and TNF might modulate the delicate balance between the anabolism and catabolism of IVDs. This could contribute to the narrow effective range of the cytokine inhibitors on the ECM anabolism of IVDs. To treat inflammatory diseases, including rheumatoid arthritis and Crohn’s disease, biopharmaceutical drugs targeting TNF, IL-1, and IL-6 provided satisfactory outcomes [34,35]. Future research on IVD degeneration in these patients using biopharmaceutical drugs may reveal the clinical feasibility of anti-cytokine drugs for IVD degeneration-related diseases.

## 4. Material and Methods

### 4.1. Cell Preparation and Culture Conditions of Alginate Beads and Explants

**Figure 7:**
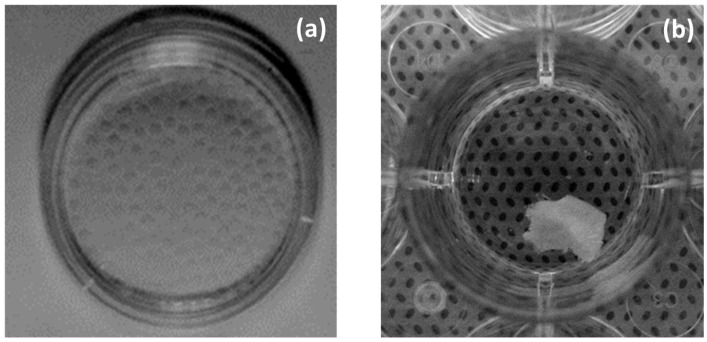
Alginate beads just made (**a**), and explant coated with 1.2% alginate (**b**).

Thirty-five human cadaveric lumbar spines (20–75 years old, average: 57.5 years old) were obtained from a regional explant bank within 24 h of death and were graded by MRI (Thompson grade 2–5) [36]. The nucleus pulposus (NP) and anulus fibrosus (AF) were isolated separately. Briefly, the outermost layer of the AF (approximately 0.5 mm) was sharply dissected and discarded to prevent contamination by cells from ligaments surrounding the IVD. The NP and AF were separated, and cells of 15 cadavers were isolated from NP and AF explants by sequential enzymatic digestion with 0.4% pronase (EMD Bioscience, La Jolla, CA, USA) for 1 h and 0.025% collagenase P (Roche Applied Science, Indianapolis, IN) and 0.001% deoxyribonuclease 2 (Sigma-Aldrich, St. Louis, MO) for 16 h in a 5% CO_2_ incubator at 37 °C. The cells were encapsulated in a 1.2% low-viscosity sterile pharmaceutical-grade alginate solution (Keltone LV, a gift from ISP Alginate Inc., San Diego, CA) at 2 × 10^6^ cells/mL (Figure 7a) [37]. The explants of 15 cadavers were cut into 5 × 5 mm pieces and coated with 1.2% alginate (Figure 7b). The alginate beads (nine beads per well in a 24-well plate) and explants were cultured in complete medium [Dulbecco’s modified Eagle’s medium and Ham’s F-12 medium (Mediatech, Herndon, VA) containing 10% fetal bovine serum (Hyclone, Logan, UT), 25 µg/mL ascorbic acid (Sigma Chemical, St. Louis, MO, USA), 360 µg/mL L-glutamine (Mediatech, Herndon, VA), and 50 µg/mL gentamicin (Invitrogen, Carlsbad, CA)], with daily medium changes. After 2 days of preculture, the beads and explants were cultured for another 3 days in complete medium in the absence or presence of IL-1 receptor antagonist (IL-1Ra, gift from Amgen, CA) at 0.1, 0.5, and 1 µg/mL, soluble TNF receptor-1 (sTNFR1, a gift from Amgen) at 0.1, 0.5, and 1 µg/mL, or both. On day 3 of treatment, the beads were dissolved in a dissolving buffer consisting of 0.15 M NaCl, 30 mM ethylenediaminetetraacetic acid, and 55 mM sodium citrate at 4 °C for 20 min with gentle shaking. The two compartments [cell-associated matrix (CM) and further removed matrix (FRM)] were then separated by mild centrifugation [38]. The explants coated with alginate were dissolved in a dissolving buffer, and the wet and dry weights were measured. 

### 4.2. DNA Content

To assess the DNA content in explants, a Quant-iT PicoGreen dsDNA Assay Kit (Invitrogen, Carlsbad, CA) was used in accordance with the manufacturer’s instructions. The DNA content was used to normalize values obtained from the following analyses. 

### 4.3. Proteoglycan (PG) Synthesis 

To assess the amount of newly synthesized PG, beads and explants were treated with ^35^S-sulfate (final concentration: 20 µCi/mL; PerkinElmer Life and Analytical Sciences, Boston, MA) for the last 4 and 16 h of culture, respectively, on day 3. At the end of the culture, the medium was collected, and the beads and explants were dissolved, as described above. The CM, FRM, and explants were digested with papain, and the amount of radiolabeled ^35^S-PGs in the digests was quantified by a rapid filtration assay following the precipitation of glycosaminoglycans with Alcian blue [39]. 

### 4.4. Collagen Synthesis 

The incorporation of radiolabeled ^3^H-proline into pepsin-resistant proteins was measured as an indicator of collagen synthesis [40]. During the last 16 h of culture, beads were radiolabeled with L-[2,3,4,5-^3^H]-proline (final concentration: 50 µCi/mL; Amersham Biosciences Corp., Piscataway, NJ). CM, FRM, and medium fractions were digested with 100 µg/mL pepsin (Sigma Chemical, St. Louis, MO) in 0.5 M acetic acid. Each sample was mixed with 30% trichloroacetic acid (final concentration: 25%; Sigma) to form a precipitate. The radioactivity of pepsin-resistant ^3^H-labeled proteins in the precipitate from each sample was quantified [40].

### 4.5. IL-1, TNF, and IL-6 Measurements

IL-1β, TNF, and IL-6 concentrations in conditioned media were determined by ELISAs (R&D Systems, Minneapolis, MN; eBioscience, San Diego, CA). To investigate the source of these cytokines, the cytokine concentrations were measured after treatment with 10 and 100 µM cycloheximide. Samples after a single freezing and thawing were used to measure intracellular cytokine levels in the explant culture system. 

### 4.6. Statistical Analysis

Values were presented as the mean ± standard error of results from three separate cultures of nine beads or single constructs. Two-way ANOVA with the Fisher PSLD test was used to assess PG and collagen syntheses. The regression coefficient was used to investigate correlations between IL-1β, TNF, and IL-6 and PG synthesis. The Spearman rank correlation was employed to assess correlations between the MRI grade of spinal discs and PG synthesis.

## 5. Conclusions

Our results demonstrated that simultaneous inhibition of IL-1β and TNF by IL-1Ra and sTNFR1 upregulated ECM anabolism in human IVD cells, and the interplay of IL-1β and TNF modulated the basal level of ECM anabolism. Additionally, IL-1β and TNF produced by NP and AF tissues in autocrine and/or paracrine fashions controlled the production of each cytokine via a feedback system. Simultaneous treatment with IL-1Ra and sTNFR1 may be a feasible approach to inhibit or prevent cytokine-induced IVD degeneration. 

## Figures and Tables

**Table 1:** Cytokine levels in the explant culture system. Donor information is presented in this table. IL-1β, TNF, and IL-6 concentrations in the explant culture system were very low and undetectable in some samples. Each donor produced various basal amounts of cytokines in the explant culture system during the first two days of culture. No significant relationship was observed between MRI or age and IL-1β, TNF, or IL-6. Data were obtained from eight donors. The minimum detection values were 0.125 pg/mL for IL-1β, 0.5 pg/mL for TNF, and 2 pg/mL for IL-6. ND: not detaectable.

				IL-1β/DNA (pg/μg)		TNF/DNA (pg/μg)		IL-6/DNA (pg/μg)	
Donor	Age	Sex	MRI	NP	AF	NP	AF	NP	AF
1	66	male	4	2.62	0.12	1.29	ND	5.51	20.01
2	35	male	2	2.32		ND		25.7	
3	57	female	2	ND	ND	ND	0.95	2.35	2.23
4	55	male	3	23.8	0.03	3.48	8.04	7.53	15.03
5	55	male	4	ND	0.12	0.28	2.81	ND	0.61
6	49	male	3	6.96	0.04	20.43	4.47	0.64	74.84
7	58	female	4	0.43	0.04	0.46	0.08	0.04	0.03
8	58	female	3	0.50	0.03	0.96	0.09	0.05	0.03

## Data Availability

Not applicable.

## References

[1] Meucci R.D., Fassa A.G., Faria N.M. (2015). Prevalence of chronic low back pain: Systematic review. Rev. Saude Publica.

[2] Andersson G.B. (1999). Epidemiological features of chronic low-back pain. Lancet.

[3] Yurube T., Takeoka Y., Kanda Y., Kuroda R., Kakutani K. (2023). Intervertebral disc cell fate during aging and degeneration: Apoptosis, senescence, and autophagy. N. Am. Spine Soc. J..

[4] Oegema T.R. (1993). Biochemistry of the intervertebral disc. Clin. Sports Med..

[5] Lotz J.C., Hsieh A.H., Walsh A.L., Palmer E.I., Chin J.R. (2002). Mechanobiology of the intervertebral disc. Biochem. Soc. Trans..

[6] Fontana G., See E., Pandit A. (2015). Current trends in biologics delivery to restore intervertebral disc anabolism. Adv. Drug Deliv. Rev..

[7] Molinos M., Almeida C.R., Caldeira J., Cunha C., Goncalves R.M., Barbosa M.A. (2015). Inflammation in intervertebral disc degeneration and regeneration. J. R. Soc. Interface.

[8] Miyazaki K., Miyazaki S., Yurube T., Takeoka Y., Kanda Y., Zhang Z., Kakiuchi Y., Tsujimoto R., Ohnishi H., Matsuo T. (2022). Protective Effects of Growth Differentiation Factor-6 on the Intervertebral Disc: An In Vitro and In Vivo Study. Cells.

[9] Osada R., Ohshima H., Ishihara H., Yudoh K., Sakai K., Matsui H., Tsuji H. (1996). Autocrine/paracrine mechanism of insulin-like growth factor-1 secretion, and the effect of insulin-like growth factor-1 on proteoglycan synthesis in bovine intervertebral discs. J. Orthop. Res..

[10] Shinmei M., Masuda K., Kikuchi T., Shimomura Y. (1989). The role of cytokines in chondrocyte mediated cartilage degradation. J. Rheumatol. Suppl..

[11] Pulai J.I., Chen H., Im H.J., Kumar S., Hanning C., Hegde P.S., Loeser R.F. (2005). NF-kappa B mediates the stimulation of cytokine and chemokine expression by human articular chondrocytes in response to fibronectin fragments. J. Immunol..

[12] Kato K., Akeda K., Miyazaki S., Yamada J., Muehleman C., Miyamoto K., Asanuma Y.A., Asanuma K., Fujiwara T., Lenz M.E. (2021). NF-kB decoy oligodeoxynucleotide preserves disc height in a rabbit anular-puncture model and reduces pain induction in a rat xenograft-radiculopathy model. Eur. Cells Mater..

[13] Aida Y., Maeno M., Ito-Kato E., Suzuki N., Shiratsuchi H., Matsumura H. (2004). Effect of IL-1alpha on the expression of cartilage matrix proteins in human chondrosarcoma cell line OUMS-27. Life Sci..

[14] Thompson J.P., Oegema T.R., Bradford D.S. (1991). Stimulation of mature canine intervertebral disc by growth factors. Spine.

[15] Burke J.G., Watson R.W.G., Conhyea D., McCormack D., Dowling F.E., Walsh M.G., Fitzpatrick J.M. (2003). Human nucleus pulposis can respond to a pro-inflammatory stimulus. Spine.

[16] Le Maitre C.L., Hoyland J.A., Freemont A.J. (2007). Interleukin-1 receptor antagonist delivered directly and by gene therapy inhibits matrix degradation in the intact degenerate human intervertebral disc: An in situ zymographic and gene therapy study. Arthritis Res. Ther..

[17] Le Maitre C.L., Freemont A.J., Hoyland J.A. (2005). The role of interleukin-1 in the pathogenesis of human intervertebral disc degeneration. Arthritis Res. Ther..

[18] Seguin C.A., Pilliar R.M., Roughley P.J., Kandel R.A. (2005). Tumor necrosis factor-alpha modulates matrix production and catabolism in nucleus pulposus tissue. Spine.

[19] Ghivizzani S.C., Kang R., Georgescu H.I., Lechman E.R., Jaffurs D., Engle J.M., Watkins S.C., Tindal M.H., Suchanek M.K., McKenzie L.R. (1997). Constitutive intra-articular expression of human IL-1 beta following gene transfer to rabbit synovium produces all major pathologies of human rheumatoid arthritis. J. Immunol..

[20] Luoma K., Riihimaki H., Luukkonen R., Raininko R., Viikari-Juntura E., Lamminen A. (2000). Low back pain in relation to lumbar disc degeneration. Spine.

[21] Doita M., Kanatani T., Harada T., Mizuno K. (1996). Immunohistologic study of the ruptured intervertebral disc of the lumbar spine. Spine.

[22] Shinmei M., Kikuchi T., Yamagishi M., Shimomura Y. (1988). The role of interleukin-1 on proteoglycan metabolism of rabbit annulus fibrosus cells cultured in vitro. Spine.

[23] Le Maitre C.L., Freemont A.J., Hoyland J.A. (2006). A preliminary in vitro study into the use of IL-1Ra gene therapy for the inhibition of intervertebral disc degeneration. Int. J. Exp. Pathol..

[24] Hosaka K., Ryu J., Saitoh S., Ishii T., Kuroda K., Shimizu K. (2005). The combined effects of anti-TNFalpha antibody and IL-1 receptor antagonist in human rheumatoid arthritis synovial membrane. Cytokine.

[25] Kobayashi M., Squires G.R., Mousa A., Tanzer M., Zukor D.J., Antoniou J., Feige U., Poole A.R. (2005). Role of interleukin-1 and tumor necrosis factor alpha in matrix degradation of human osteoarthritic cartilage. Arthritis Rheum..

[26] Nixon R., Bansback N., Brennan A. (2007). The efficacy of inhibiting tumour necrosis factor alpha and interleukin 1 in patients with rheumatoid arthritis: A meta-analysis and adjusted indirect comparisons. Rheumatology.

[27] Horai R., Asano M., Sudo K., Kanuka H., Suzuki M., Nishihara M., Takahashi M., Iwakura Y. (1998). Production of mice deficient in genes for interleukin (IL)-1alpha, IL-1beta, IL-1alpha/beta, and IL-1 receptor antagonist shows that IL-1beta is crucial in turpentine-induced fever development and glucocorticoid secretion. J. Exp. Med..

[28] Clements K.M., Price J.S., Chambers M.G., Visco D.M., Poole A.R., Mason R.M. (2003). Gene deletion of either interleukin-1beta, interleukin-1beta-converting enzyme, inducible nitric oxide synthase, or stromelysin 1 accelerates the development of knee osteoarthritis in mice after surgical transection of the medial collateral ligament and partial medial meniscectomy. Arthritis Rheum..

[29] Geng Y., Valbracht J., Lotz M. (1996). Selective activation of the mitogen-activated protein kinase subgroups c-Jun NH2 terminal kinase and p38 by IL-1 and TNF in human articular chondrocytes. J. Clin. Investig..

[30] Fukui N., Zhu Y., Maloney W.J., Clohisy J., Sandell L.J. (2003). Stimulation of BMP-2 expression by pro-inflammatory cytokines IL-1 and TNF-alpha in normal and osteoarthritic chondrocytes. J. Bone Jt. Surg. Am..

[31] Studer R.K., Gilbertson L.G., Georgescu H., Sowa G., Vo N., Kang J.D. (2008). p38 MAPK inhibition modulates rabbit nucleus pulposus cell response to IL-1. J. Orthop. Res..

[32] Ulfgren A.K., Andersson U., Engstrom M., Klareskog L., Maini R.N., Taylor P.C. (2000). Systemic anti-tumor necrosis factor alpha therapy in rheumatoid arthritis down-regulates synovial tumor necrosis factor alpha synthesis. Arthritis Rheum..

[33] Williams R.O., Feldmann M., Maini R.N. (2000). Cartilage destruction and bone erosion in arthritis: The role of tumour necrosis factor alpha. Ann. Rheum. Dis..

[34] Hanauer L.B. (2002). Comment on prescribing tumor necrosis factor inhibitors. Arthritis Rheum..

[35] Klareskog L., Catrina A.I., Paget S. (2009). Rheumatoid arthritis. Lancet.

[36] Thompson J.P., Pearce R.H., Schechter M.T., Adams M.E., Tsang I.K., Bishop P.B. (1990). Preliminary evaluation of a scheme for grading the gross morphology of the human intervertebral disc. Spine.

[37] Masuda K., Takegami K., An H., Kumano F., Chiba K., Andersson G.B.J., Schmid T., Thonar E. (2003). Recombinant osteogenic protein-1 upregulates extracellular matrix metabolism by rabbit annulus fibrosus and nucleus pulposus cells cultured in alginate beads. J. Orthop. Res..

[38] Chiba K., Andersson G.B., Masuda K., Thonar E.J. (1997). Metabolism of the extracellular matrix formed by intervertebral disc cells cultured in alginate. Spine.

[39] Mok S.S., Masuda K., Hauselmann H.J., Aydelotte M.B., Thonar E.J. (1994). Aggrecan synthesized by mature bovine chondrocytes suspended in alginate. Identification of two distinct metabolic matrix pools. J. Biol. Chem..

[40] Koyano Y., Hammerle H., Mollenhauer J. (1997). Analysis of 3H-proline-labeled protein by rapid filtration in multiwell plates for the study of collagen metabolism. Biotechniques.

